# From By‐Products to Flavored Oils: Ultrasound‐Assisted Enrichment of Refined Olive and Grape Seed Oils and Consumer Perception Under Blind and Informed Conditions

**DOI:** 10.1002/fsn3.72374

**Published:** 2026-09-17

**Authors:** Samanta Corsetti, Lucia Irene Bailetti, Sofia Calzolari, Monia Floridi, Gianni Sagratini, Laura Alessandroni

**Affiliations:** ^1^ School of Pharmacy, Chemistry Interdisciplinary Project (CHIP) University of Camerino Camerino Italy; ^2^ New Flavours Srl, Zona Industriale Gioiello Monte Santa Tiberina Italy

**Keywords:** by‐product valorization, consumer perception, grape seed oil, refined olive oil, ultrasound‐assisted extraction

## Abstract

The sustainable valorization of underutilized oils and plant by‐products requires not only technological upgrading but also consumer acceptance. In this study, basil and chili pepper by‐products were used to flavor refined olive oil and grape seed oil through ultrasound‐assisted extraction (UAE). The resulting oils were characterized in terms of quality parameters, antioxidant activity, total phenolic content, and total carotenoid content to assess the overall enrichment achieved by UAE, while consumer perception was investigated under both blind and informed conditions. UAE effectively enhanced the bioactive profile of both refined olive and grape seed oils, yielding up to a twofold increase in total phenolic content compared with the corresponding unflavored oils. Chili pepper‐ and basil‐flavored grape seed oils exhibited the highest antioxidant activity, reaching 3.9 μmol TE/g oil, whereas oxidative changes were influenced more by the flavoring matrix than by the lipid carrier. Sensory evaluation under blind conditions identified opportunities for product optimization, particularly by increasing basil aroma intensity and moderating chili pungency, as highlighted by Just‐About‐Right (JAR) and penalty analyses. More importantly, providing consumers with information about the enrichment process significantly improved product acceptance. Flavor liking and purchase intention increased under informed conditions, with improvements of up to 20.37% for basil‐flavored grape seed oil. Considering that 77.14% of participants were unfamiliar with grape seed oil, these findings demonstrate that combining sustainable technological upgrading with effective consumer communication can substantially enhance the acceptance and market potential of underutilized lipid matrices and food industry by‐products.

## Graphical abstract

Underutilized refined olive and grape seed oils were upgraded by ultrasound‐assisted aromatization with basil and chili pepper by‐products. UAE enhanced their bioactive profile, with up to a twofold increase in total phenolic content. Consumer testing highlighted opportunities to optimize aroma and pungency, while information on the enrichment process significantly improved product acceptance and purchase intention, particularly for the unfamiliar grape seed oil. These findings support UAE as a sustainable strategy for valorizing underused oils and food by‐products.

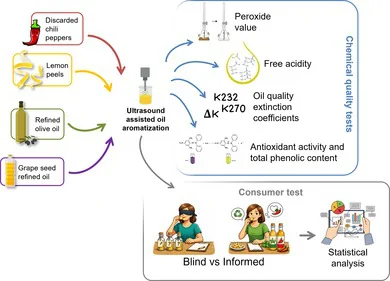

## 1 Introduction

Vegetable oils play a central role in the human diet, and global consumption has progressively shifted toward seed oils, which currently account for almost all vegetable oil use worldwide. Among these, some oils remain relatively underutilized despite their technological potential. Grape seed oil (GSO), for instance, is a secondary product of the wine industry obtained from grape pomace and subsequently refined before commercialization (Delgado and Guinard 2011). Similarly, during extra virgin olive oil (EVOO) production, a considerable proportion of olive oils fail to meet the quality requirements for EVOO classification because of defects or excessive acidity, with approximately 41% of collected oils being downgraded (Cecchi et al. 2016). To reduce economic losses, these oils are refined and blended with virgin oils, generating lower‐cost commercial products with reduced sensory complexity.

Although refining is essential for the recovery and stabilization of these matrices, it also removes many compounds responsible for aroma, flavor, and bioactive properties, resulting in oils with a neutral sensory profile and lower antioxidant potential (Civan and Kumcuoglu 2019; Soares et al. 2020). In this context, aromatization represents a promising strategy to improve both the sensory quality and market value of refined oils. When combined with the use of food industry by‐products, this approach also aligns with circular economy principles by simultaneously valorizing low‐value lipid matrices and discarded plant materials.

From a technological perspective, oil aromatization can be considered an extraction process in which the oil acts as a solvent for lipophilic aromatic and bioactive compounds. Among green extraction technologies, ultrasound‐assisted extraction (UAE) has attracted increasing interest because of its reduced extraction times, lower solvent and energy requirements, and improved extraction efficiency (Soares et al. 2020). Previous studies have mainly focused on the chemical and technological characterization of flavored oils, particularly virgin and extra virgin olive oils (Civan and Kumcuoglu 2019; Soares et al. 2020). However, limited attention has been paid to the sustainable upgrading of underutilized oils such as refined olive oil and grape seed oil through UAE‐based aromatization strategies using plant by‐products.

Moreover, although the enrichment of oils with bioactive compounds has been widely investigated, improvements in chemical composition do not necessarily translate into consumer acceptance. Consumer perception of flavored oils is influenced not only by intrinsic sensory characteristics but also by extrinsic factors such as familiarity, origin, processing information, sustainability claims, and perceived health benefits (Delgado and Guinard 2011; Caporale et al. 2006). This aspect may be particularly relevant for refined oils, which are often perceived as lower‐quality products, and for grape seed oil, which remains unfamiliar to many consumers.

According to expectation–disconfirmation theory, information provided before tasting may shape sensory expectations and subsequently influence hedonic evaluation through assimilation or contrast effects (McSweeney 2022; Latino et al. 2022). Positive information related to sustainability, functional enrichment, or by‐product valorization may induce consumers to adjust their perception toward the expected benefits, especially when evaluating unfamiliar or underutilized products. Therefore, investigating flavored oils under both blind and informed conditions may provide valuable insights into the role of information disclosure in shaping liking and purchase intention (Kalua et al. 2007; Cecchi et al. 2021).

To date, the combined evaluation of chemical enrichment, oil quality, and consumer perception following UAE aromatization remains poorly explored, particularly for refined olive oil and grape seed oil used as lipid carriers (Nardella et al. 2021). In addition, studies integrating blind and informed sensory evaluation with sustainability‐related information are still scarce in the scientific literature.

Therefore, the novelty of the present study lies in the integrated evaluation of the ultrasound‐assisted aromatization of refined olive oil and grape seed oil using basil and chili pepper by‐products, combining chemical characterization with consumer perception under both blind and informed conditions. While previous studies have mainly focused on the technological or chemical properties of flavored oils, little attention has been devoted to understanding whether the enrichment achieved through sustainable processing strategies translates into consumer acceptance, particularly for underutilized lipid matrices (Soares et al. 2020; Civan and Kumcuoglu 2019; Corsetti et al. 2026). This aspect is especially relevant because products obtained from circular economy approaches frequently face market barriers related to unfamiliar sensory profiles and limited consumer awareness, making sensory validation essential for their successful commercialization. Furthermore, by comparing blind and informed evaluations, this study investigates how information related to sustainability and bioactive enrichment influences consumer responses, providing insights into the role of expectation‐driven perception in the acceptance of sustainable flavored oils.

Accordingly, the aim of this study was to investigate the feasibility of ultrasound‐assisted extraction as a green strategy to valorize refined olive oil and grape seed oil through aromatization with basil and chili pepper by‐products. The flavored oils were characterized in terms of antioxidant activity, total phenolic and carotenoid contents, and conventional quality parameters, while consumer perception was assessed through blind and informed sensory evaluations combined with Just‐About‐Right (JAR) and penalty analyses. This integrated approach provides both technological and consumer‐oriented evidence supporting the development of sustainable, value‐added flavored oils from underutilized food industry side streams.

## 2 Materials and Methods

### 2.1 Samples Collection

Three same‐batch bottles of grape seed oil (GSO) and three same‐batch bottles of olive oil (OO) were purchased from a local supermarket in Camerino (MC, Italy) from two different commercial brands. All flavored oils were prepared from these batches to ensure experimental consistency. Oils were all stored in dark bottles always at room temperature (25°C ± 0.5°C) and protected from light. According to the label, GSO was refined and obtained by solvent extraction and OO was a mixture of refined and virgin olive oils, according to Reg. (EU) 2022/2104.

Chili peppers were selected among those Calabrian chili peppers ( *Capsicum Annuum* L., Naso Di Cane subvariety) which were evaluated as not suitable for direct sale, due to the size or shape requirements. Also, the basil leaves ( *Ocimum basilicum* L.) were selected among those not conforming to high standards‐PDO Genoese basil supply chain. New Flavours company (Via dell'Artigianato 7, Monte Santa Tiberina, PG, Italy) provided samples of whole chili peppers (seeds, pulp and skin included) and basil leaves. Both materials were dried in an oven at 40°C until constant weight and finely ground using a Waring Blender LB20 EG (Waring, CT, USA) before use.

### 2.2 Vegetable Oils Aromatization Step

Samples from each one of the three bottles of OO and the other three from GSO and aromatization materials were collected by New Flavours company. The flavoring step was performed by the company according to a previously published optimized method (Corsetti et al. 2026) through a 38 kHz probe‐type ultrasonic system equipped with 70% of a 1000 W nominal power generator (Unitech srl). The parameters were the following ones: a temperature of 35°C and a ratio of 0.2 g/mL for 10 min for chili pepper and a temperature of 30°C and a ratio of 0.15 g/mL for 10 min for basil aromatization, with the temperature during the process being automatically controlled through a thermostatic cooling system. Three independent extraction batches were carried out for each flavoring matrix (chili pepper or basil), using every time 500 mL from a different batch of OO or GSO as solvent. Thus, the nominal power density, calculated as nominal power divided by extraction volume, was 1.4 W mL^−1^. After UAE, solid material was filtered through a cartridge filter (5 μm nominal pore size) and chili pepper‐flavored olive oil and grape seed oil (Cpep‐OO or Cpep‐GSO) were obtained, along with basil‐flavored olive oil and grape seed oil (Bas‐OO or Bas‐GSO).

### 2.3 Free Acidity, Specific Extinction Coefficients and Peroxide Value Analyses

Free acidity (FFA%), specific extinction coefficients (K232, K270, and ΔK) and the peroxide value (POV; expressed as mEq O_2_/Kg oil) determination were conducted according to the protocols of EC Reg. no. 2568/1991 and the subsequent amendments reported by the European Commission (European Commission 2016). Analyses were performed on OO, GSO, Cpep‐OO, Cpep‐GSO, Bas‐OO and Bas‐GSO. Shortly, 5 g of each oil were dissolved in an diethyl ether/ethanol solution (1:1 v/v), and FFA was measured through alkalimetric titration with a 0.05 mol/L KOH ethanolic solution using 1% ethanolic phenolphthalein as indicator. POV analyses were achieved dissolving 5 g of sample in chloroform and acetic acid and performing iodometric titration with sodium thiosulfate 0.01 mol/L, using starch indicator. Each sample (i.e., each one of the three unflavored OO or GSO collected from the bottles with different batch numbers and each related flavored sample) was analyzed in triplicate, as well the specific extinction coefficients, which were evaluated spectrophotometrically, according to the aforementioned Regulation. All solvents and reagents were purchased from Merck (Milan, Italy). Then a one‐way ANOVA statistical analysis was performed, followed by Tukey's HSD post hoc test. Since three independent extractions were performed for each oil sample and each extract was analyzed in triplicate for analytical accuracy, *n* was equal to 3 independent extractions for all chemical analyses in the statistical analysis (the mean value of the three results of analytical replicates was used for statistics).

### 2.4 Total Phenolic Content, Radical‐Scavenging Activity and Total Carotenoid Content of Flavored Oils and Related Control Samples

For total phenolic content (TPC) analysis, the procedure reported by Ricciutelli et al. (2017) was involved. Briefly, 2.5 g of sample was diluted 1:1 v/v in hexane and 2.5 mL of MeOH/H_2_O 80:20 v/v mixture was used to extract phenolic molecules. After centrifugation at 5000 rpm for 5 min, the polar fraction was collected. The procedure was repeated three times and the collected samples were washed twice with hexane. Then, 2.5 mL of Folin–Ciocalteu reagent (FC) and 2.5 mL of 7.5% w/v Na_2_CO_3_ aqueous solution were added. The solution volume was brought to 50 mL with distilled water and placed in the dark for 4 h at room temperature. The absorbance was then measured at 765 nm with Cary 8454 UV–visible spectrophotometer (Agilent Technologies, Woburn, MA, USA). The calibration curve was prepared with gallic acid and results are expressed as mg of gallic acid equivalents (GAE)/Kg of oil.

Antioxidant activity was measured through the DPPH assay (Alessandroni et al. 2024; Corsetti et al. 2026): 0.8 mL of oil was mixed with 2.4 mL of methanol, vortexed and centrifuged for 3 min, collecting the upper methanol phase. Subsequently, 4.5 mL of DPPH (2,2‐diphenyl‐1‐picrylhydrazyl radical) 0.1 mM ethanolic solution was mixed with 0.5 mL of methanolic extract. Then samples were stored for 30 min in the dark at room temperature and the absorbance was read at 517 nm. For the calibration curve, Trolox was used as the reference antioxidant compound and the results were expressed in μmol Trolox‐equivalents (TE)/g oil.

The Total Carotenoid Content (TCC) (expressed as μg/g of dry extract) was measured on 30 mg of oil dissolved in 5 mL of hexane and calculated through the following Equation after directly measuring the absorbance at 450 nm.

$$\mathrm{TCC}\;\left(\mathrm{\mu g}/g\right)=\frac{A\times V\left(\mathrm{mL}\right)\times 10^{4}}{{A_{1}}_{\mathrm{cm}}^{1\%}\times P\left(g\right)}$$

where A is the sample absorbance, V (mL) is the total volume of the extract, ${A_{1}}_{\mathrm{cm}}^{1\%}$ is the β‐carotene extinction coefficient in petroleum ether (2592 mL/g·cm) and P (g) is the weight of the dried extract (Larocca et al. 2023).

Similarly to what has been reported in Section 2.3, each assay was repeated in triplicate for each unflavored oil and each chili pepper/basil‐flavored counterpart with the same statistical approach, using the one‐way ANOVA and Tukey's HSD post hoc test. TPC and antioxidant activity were assessed as global indicators of phenolic‐related quality. All solvents and reagents were purchased from Merck (Milan, Italy).

### 2.5 Blind and Informed Consumer Tests

A total of 70 untrained consumers were recruited on a voluntary basis (35.71% males and 64.29% females, 99% Italian). The privacy rights have been observed and a written informed consent was obtained from all the participants before the study. The consumer study involved voluntary participation and anonymous data collection using food products intended for human consumption and produced by New Flavours company (Via dell'Artigianato 7, Monte Santa Tiberina, PG, Italy). According to the applicable institutional guidelines, formal ethical committee approval was not required for this type of non‐invasive sensory evaluation. Informed consent was obtained from all participants prior to the study.

To prepare participants for the evaluation process, a preliminary practice session was held. During this session, the purpose and methodology of the test were explained, including a demonstration of the 9‐point hedonic scale and examples of sensory attributes to be evaluated. This step was essential to ensure a uniform understanding of the assessment criteria (Branciari et al. 2019).

Consumer test was performed at the University of Camerino. The test was conducted in a white environment and in a different location from the place of sample preparation, under artificial daylight‐type illumination, air circulation, temperature control (between 22°C and 24°C), and assuring sample homogeneity, according to ISO 8589:2007.

A 3‐digit randomized code was assigned to each disposable food‐grade plastic cup, where 8 mL of each oil sample were served, according to ISO 6658. The questionnaire was administered by scanning a QR code, allowing the consumer to be guided step by step for each activity requested. The questionnaire was divided into several sections, detailed in Tables S1, S2 and S3. First, the Blind test section has been carried out, using the same set of questions for both basil and chili pepper‐flavored oils; the samples order was set to avoid possible desensitization caused by their pungency. Consumers were asked to evaluate the appearance (color), the odor and the flavor of each sample, using a 9‐point hedonic scale, (score 1 for “I don't like it at all” and 9 for “I like it very much”) (Table S1). Then the intensities of the odor and flavor as well as the pungency for the latter, using a 5‐point hedonic rating scale (a score of 3 represents the right intensity, a score of 1 a too low intensity and 5 a too intense attribute) (Table S2).

The last section consisted of an Informed Expectation test. At this point consumers received information about the type of oil, aromatization matrix, total polyphenol content, and antioxidant activity of the samples they were testing. Thus, basil‐flavored GSO and OO were administrated to investigate the influence of the information on the general liking and flavor liking (Table S3). The informed consumer test was performed only on basil‐flavored oils to investigate the effect of extrinsic informative cues and the specific effect of bioactive‐related information on consumer perception. Chili‐flavored oils were excluded from this test to avoid potential sensory adaptation and TRPV1 receptor desensitization induced by repeated exposure to capsaicinoids. The informed evaluation was conducted immediately after the blind test within the same session to directly assess the effect of information disclosure on the perception of the same samples while minimizing inter‐session variability. Thus, the order of administration of the samples was randomized, except for the fact that chili samples were administered last, after the Blind Test and subsequently the Informed Test were completed for the basil‐flavored oils. To reduce sensory fatigue and all the carry‐over effects, unsalted crackers for palate cleansing procedures and breaks were applied between all tastings, and the number of evaluated samples was limited.

Statistical differences in overall and attribute‐specific liking scores were assessed by one‐way ANOVA followed by Tukey's HSD post hoc test (*n* was equal to the 70 answers of the consumers for the statistical analysis). The penalty analysis tool of XLSTAT software (version 2023.1.4.1408) was used to determine the proportion of consumers rating each attribute intensity as JAR (“Just About Right”) or as “too much”/“too low”, and to calculate the corresponding mean drops in overall liking for the latter categories (the 20% of people reporting the attribute intensity was the minimal threshold considered).

## 3 Results and Discussion

### 3.1 Qualitative Parameters of the Flavored and Unflavored Oils

The quality parameters of flavored and unflavored oils were evaluated to assess the effects of flavoring on oil oxidation and overall chemical quality. Table 1 reports the results of peroxide value (PV), free fatty acids (FFA), and UV spectrophotometric indices (K232, K270, and ΔK).

**TABLE 1:** Oil quality parameters results. Different superscript letters indicate statistically significant differences detected through one‐way ANOVA followed by Tukey's HSD post hoc test. OO and GSO stand for olive and grape seed oil, respectively, while Cpep or Bas are the abbreviations for chili pepper or basil‐flavored oils.

Samples	POV (meqO_2_/kg)	FFA (% oleic acid)	K232	K270	ΔK
OO	3.25 ± 0.35^a^	0.13 ± 0.02^a^	2.01 ± 0.22^a^	0.89 ± 0.13^a^	0.09 ± 0.01^a^
GSO	5.75 ± 1.06^b^	0.29 ± 0.01^c^	3.74 ± 0.43^c^	2.64 ± 0.46^b^	0.39 ± 0.06^c^
Cpep‐OO	3.10 ± 0.57^ab^	0.36 ± 0.07^cd^	2.92 ± 0.51^bc^	0.99 ± 0.21^a^	0.10 ± 0.01^a^
Cpep‐GSO	4.25 ± 0.35^ab^	0.47 ± 0.09^d^	3.62 ± 0.58^c^	2.75 ± 0.20^b^	0.38 ± 0.005^c^
Bas‐OO	13.25 ± 1.41^c^	0.21 ± 0.01^b^	2.68 ± 0.35^b^	1.01 ± 0.25^a^	0.13 ± 0.02^b^
Bas‐GSO	14.75 ± 1.42^c^	0.25 ± 0.05^bc^	3.36 ± 0.30^c^	2.64 ± 0.06^b^	0.40 ± 0.002^c^

In European Union, these oil quality parameters are regulated by the Reg. (EU) 2022/2104 for olive oils, and by the Codex Alimentarius Standard For Named Vegetable Oils (CXS 210‐1999) for grape seed oils (European Union 2022). In fact, these parameters are commonly used as quality indicators in the literature (Bozan and Temelli 2008; Knazicka et al. 2025; Odeh et al. 2021). They provide information on the oxidative status, hydrolytic changes and general chemical stability of oils. Their evaluation is therefore useful for assessing quality variations associated with processing and flavoring (Lamas et al. 2022; Díaz‐Montaña et al. 2023).

For both OO and GSO samples, peroxide value remained statistically unchanged after chili pepper flavoring by UAE. In contrast, basil flavoring led to a significant PV increase, with mean values of 3.10 ± 0.57 and 4.25 ± 0.35 mEq O_2_/kg for chili pepper‐flavored OO and GSO, respectively, compared to 13.25 ± 1.41 and 14.75 ± 1.42 mEq O_2_/kg observed in the corresponding basil‐flavored oils. A similar trend was experienced by Odeh et al. (2021) on both basil and chili‐flavored cold‐pressed flaxseed oils.

As previously mentioned, these standards are measured solely as reference values for quality comparison rather than as legal limits (Lamas et al. 2022; Díaz‐Montaña et al. 2023). Overall, all quality parameters of the flavored OOs and GSOs examined remained below these reference values, with the only exception of the K232 value for Cpep‐OO (2.92), which exceeded the limit set for this category of olive oil (Reg. (EU) 2022/2104), while K270 (0.99) sits close to its threshold. Besides, it's also relevant to underline that especially the peroxide value Bas‐GSO particularly exceeded the POV threshold suggested by the Codex Standard for Named Vegetable Oils (CXS 210‐1999) for unflavored refined seed oils (acidity < 0.6 mg KOH/g; POV < 10 mEq O_2_/kg) (Odeh et al. 2021).

This agrees with the findings of other authors conducting similar analysis of an infusion flavored virgin olive oil (Caponio et al. 2016). For comparison, Caporaso et al. (2013) investigated these quality parameters in refined olive oil infused with chili pepper ( *Capsicum annuum* L., 20% w/w) for 6 days at room temperature. They reported a sevenfold increase in FFA, more than a twofold rise in PV, and increases of approximately 40% and 60% in K232 and K270, respectively, compared with the control oil. In this regard, an important consideration is treatment time. A longer treatment exposes the oil to oxidative agents for a longer period, resulting in higher FFA and PV values, regardless of the type of flavoring used. In this study, times were reduced to 10 min for chili pepper and 10 min basil aromatization treatments.

In conclusion, the initial peroxide value, free fatty acid content, and extinction coefficients of the unflavored vegetable oils differed prior to UAE flavoring, reflecting the higher susceptibility of seed oils to oxidative degradation (Knazicka et al. 2025). However, the extent of the observed changes was mainly influenced by the flavoring matrix (chili pepper or basil), rather than by the type of vegetable oil used, considering that different characteristics of these spices, including the presence of diverse endogenous enzymes or pro‐oxidant compounds, may influence that aspect. Since the study was conducted using a single commercial batch for each oil, the results should be interpreted within the characteristics of the selected products. Future studies including multiple production batches and commercial brands would further strengthen the generalizability of the findings.

### 3.2 Change in Antioxidant Activity, Phenolic and Carotenoids Contents in Flavored Oils

**FIGURE 1:**
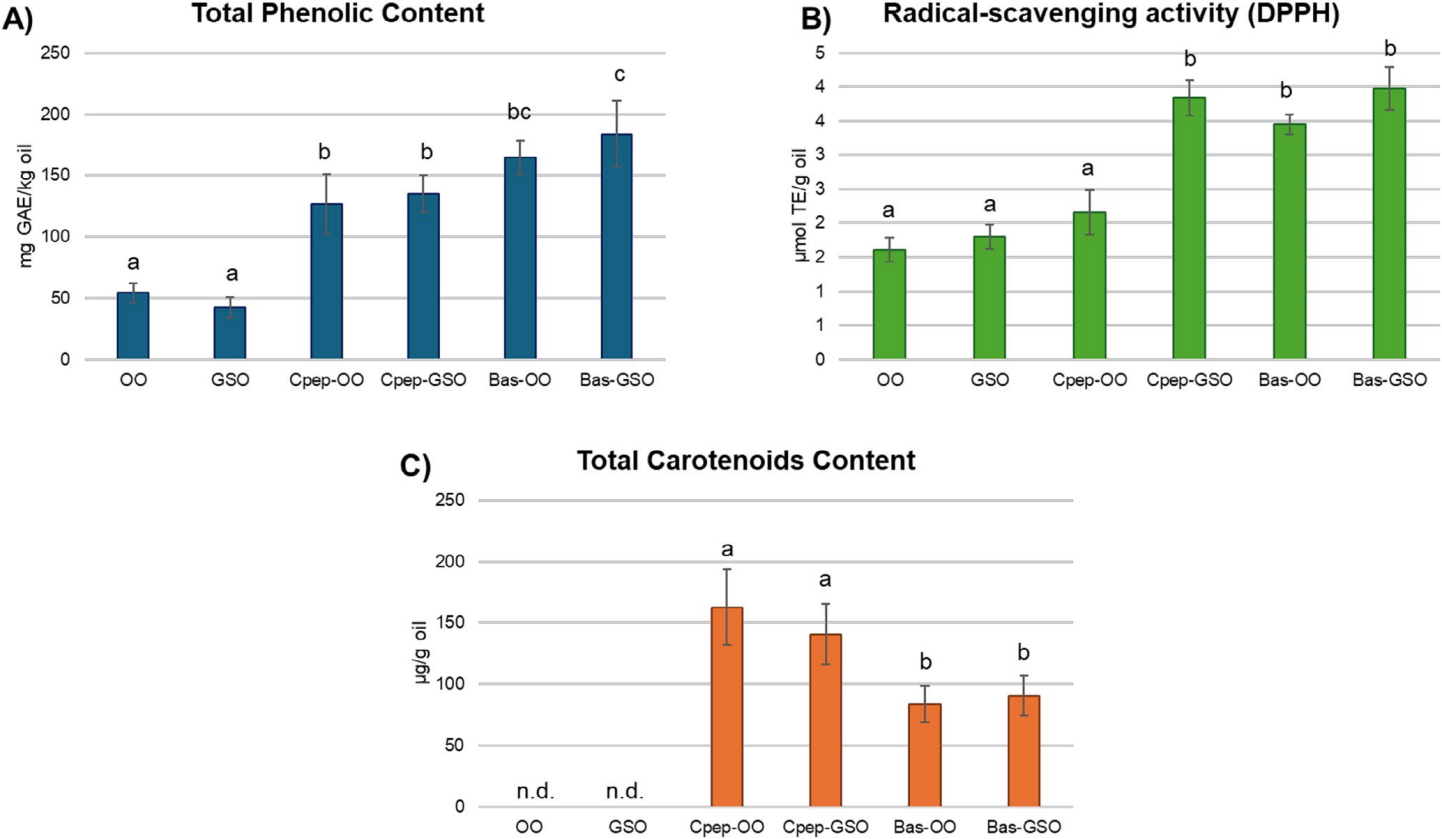
Flavored oils enrichment assessment. (A) TPC values, (B) radical scavenging activity (DPPH‐assay results), and (C) TCC values of control, chili‐ or basil‐flavored oils. OO and GSO stand for olive and grape seed oil, respectively, and Cpep or Bas are the abbreviations for chili pepper or basil‐flavored oils. Different letters show statistically significant differences among samples (one‐way ANOVA followed by Tukey's HSD post hoc test, where *n* = 70 consumers). n.d.: not detectable.

Flavoring processes may significantly influence the antioxidant activity and the concentration of bioactive compounds in edible oils. This section examines the changes in antioxidant capacity, total phenolic content, and carotenoid levels in the flavored oils. Results of the spectrophotometrically measured features are reported in Figure 1. It's relevant to underline that the DPPH assay was selected as a rapid and widely used method to compare the radical‐scavenging activity among the different flavored oil formulations since that was the aim of the study. Thus, the results of the antioxidant activity should be interpreted as an estimate of it, rather than a comprehensive assessment.

No statistically significant differences were reported between the two control samples, underlining that the refining process strongly impacts the stability of antioxidant bioactive compounds. On this line, the flavoring process revealed as an effective approach to increase this characteristic.

Both chili pepper‐flavored oils (Cpep‐OO and Cpep‐GSO) showed a statistically significant and higher TPC than unflavored ones (OO and GSO) with similar values (Figure 1A). This outline demonstrates that these oils are good carriers for polyphenols extraction from chili peppers, which are widely known as a good source of these bioactive compounds and their associated healthy properties (Baenas et al. 2018; Corsetti et al. 2025).

The TPC level of OO and its enhancement in Cpep‐OO are in line Civan and Kumcuoglu (2019)'s results, which optimized a refined olive oil UAE aromatization using the pulp of hot pepper paste. Similarly, Caporaso et al. (2013) described an important enhancement of both TPC and antioxidant activity after the aromatization of an edible OO with dried chili pepper ( *Capsicum annuum* L.) through conventional infusion. On the contrary, previously published studies on EVOO aromatization through infusions, reported a not‐increasing TPC value after treatment, which could be due to a higher phenolic presence in extra virgin olive oils (EVOO) control samples (Cecchi et al. 2025; Caponio et al. 2016; Sousa et al. 2015; Odeh et al. 2021). Although TPC is higher in basil‐flavored oils than in chili pepper‐flavored ones, the higher content of other antioxidant compounds (e.g., tocopherols, carotenoids) in chili peppers could explain also their lower levels of primary oxidation products (PVs) (Hervert‐Hernandez et al. 2010; Corsetti et al. 2025).

Both basil‐flavored oils were significantly enriched in the phenolic molecules than all the other samples, with a TPC value increasing of more than 30%, compared to chili pepper‐flavored ones. The Bas‐OO values are very similar to the unflavored EVOO ones (Soares et al. 2020; Fiorini et al. 2018).

In terms of radical scavenging activity, all flavored oils showed statistically significant higher values than the control samples in the DPPH test (Figure 1B), except Cpep‐OO ones. Considering this outcome together with TPC results, it is possible to hypothesize that several non‐polyphenolic antioxidant substances are extracted from the matrices during the process. In particular, GSO seems to be more effective in radical‐scavenging compounds extraction from chili pepper than OO, while the two oils showed similar TPC enrichment capacity. This aspect was explored by other researchers who associated it with a starting intrinsic lower level of bioactive compound leading to a higher diffusion of the fat‐soluble phenolic compounds (Knazicka et al. 2025; Caponio et al. 2016; Sousa et al. 2015; Yfanti et al. 2024).

On the contrary, the flavoring of the chili pepper had a statistically significant influence on the carotenoids content of the oils. In fact, Cpep‐OO and Cpep‐GSO showed a higher TCC than both the control samples (not detectable) and the basil‐flavored ones (Figure 1C). The ability of oils to extract carotenoids is related to the predominantly non‐polar nature of these compounds. However, refining processes can result in carotenoid losses of up to 98.6%, leading to a markedly faded oil color (Flakelar et al. 2022). The differences between chili pepper‐ and basil‐flavored samples could also be due to the carotenoids content in the starting matrices. In fact, basil contains a lower concentration than chili pepper (Crișan et al. 2024; Corsetti et al. 2025).

In general, the proposed UAE flavoring process resulted in a good strategy to increase the antioxidant activity and the phenolic molecules and carotenoids concentrations in refined oils. The presence of these bioactives is crucial not only for consumer health promotion but also for oil preservation. Besides, the increased antioxidant activity observed in the flavored oils may be associated with the transfer of bioactive compounds, from different chemical classes, which are naturally present in basil and chili pepper. Indeed, basil is particularly rich in phenolic acids and antioxidant compounds, including rosmarinic acid, caffeic acid, eugenol, and linalool, which are known for their radical scavenging activity (Romano et al. 2022). Similarly, chili pepper contains not just phenolic compounds but also capsaicinoids, with recognized antioxidant properties, which may have contributed to the enhanced antioxidant capacity of the enriched oils (Materska and Perucka 2005).

Accordingly, as previously reported in literature, the acoustic cavitation phenomena during UAE promotes the release and diffusion of phenolic compounds and other bioactives from plant matrices into the solvent through an enhanced mass transfer, after the cell wall rupture (Chemat et al. 2017). Similar and enhanced UAE‐related improvements in the recovery of antioxidant and bioactive compounds from plant materials have been previously reported in edible oil systems, as well (Tiwari 2015; Soares et al. 2020; Civan and Kumcuoglu 2019).

### 3.3 Consumer Evaluation Under Blind Conditions

**FIGURE 2:**
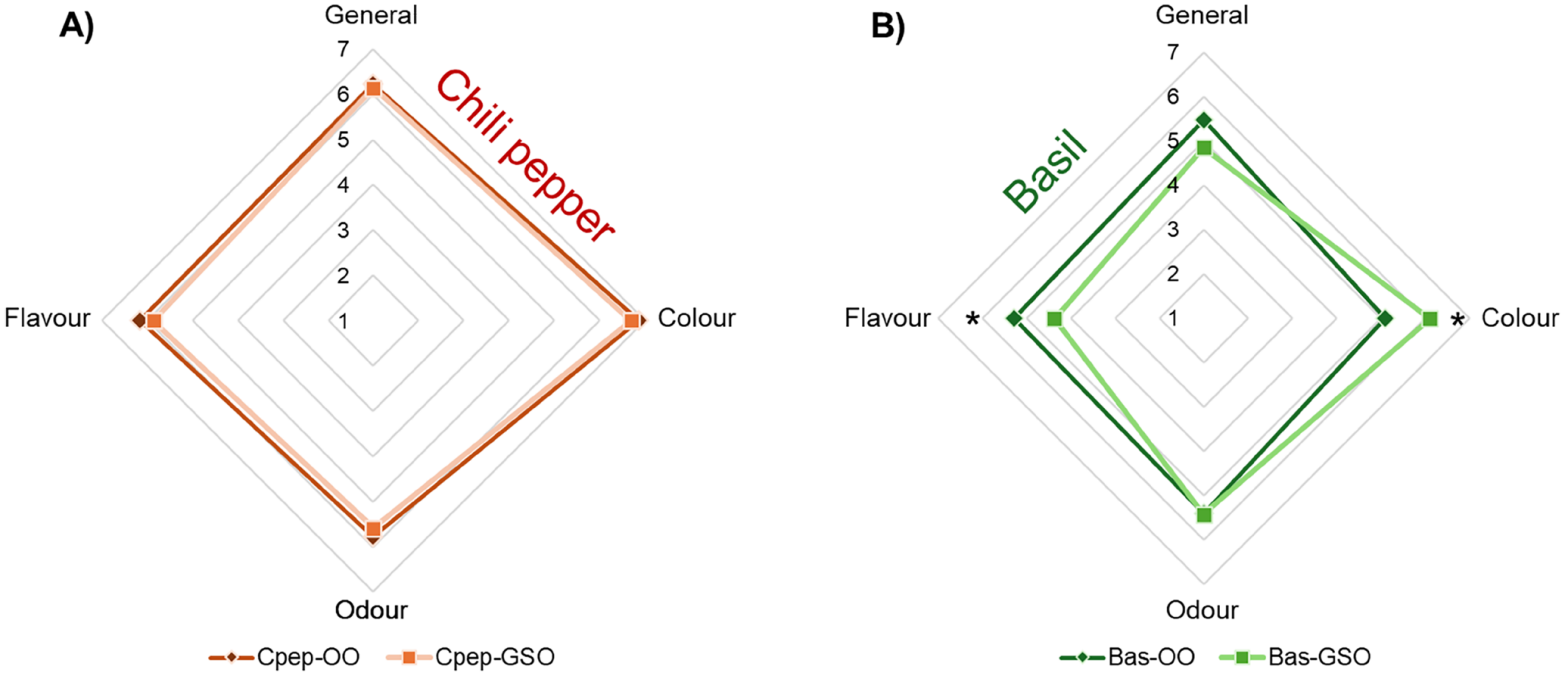
Spider plots of intensity parameters scores for chili pepper‐flavored (A) and basil‐flavored oils (B) from consumer blind test. OO and GSO stand for olive and grape seed oil, respectively, and Cpep or Bas are the abbreviations for chili pepper or basil‐flavored related oils. Asterisks indicate a statistically significant difference between the flavored‐OO and the flavored‐GSO (one‐way ANOVA followed by Tukey's HSD post hoc test, where *n* = 70 consumers).

Under blind conditions, consumers evaluated the flavored oils exclusively on the basis of their intrinsic sensory characteristics. Overall, chili‐flavored samples were characterized by higher perceived intensity scores for pungency and flavor, whereas basil‐flavored oils showed greater variability in flavor intensity perception depending on the lipid matrix. The results of evaluated sensory attributed hedonic scores have been reported in Figure 2.

The chili pepper oils were strongly appreciated by consumers, more basil‐flavored ones with general liking average scores of 6.23 and 6.14 (Cpep‐OO and Cpep‐GSO, respectively) versus 5.49 and 4.86 (Bas‐OO and Bas‐GSO, respectively).

In general, no statistically significant differences emerged from chili pepper‐flavored GSO and OO, according to consumers' scores (Figure 2A). A different scenario was reported by basil‐flavored samples where a statistically significant increase in color and flavor hedonic scores was observed for GSO and OO, respectively (Figure 2B). This finding is consistent with previously published research that hypothesized that basil's “herbaceous” notes are more in line with, or enhance, the expected “herbaceous” attributes of olive oil (Sánchez‐Rodríguez et al. 2019).

In the case of grape seed oil (GSO), the unfamiliarity of the product category did not result in significantly lower overall liking scores compared to refined olive oil (OO), suggesting that intrinsic sensory performance may compensate for limited prior knowledge when no extrinsic cues are provided. Results of preliminary habits questions are reported in Figure S1.

### 3.4 Just‐About‐Right (JAR) and Penalty Analysis

**FIGURE 3:**
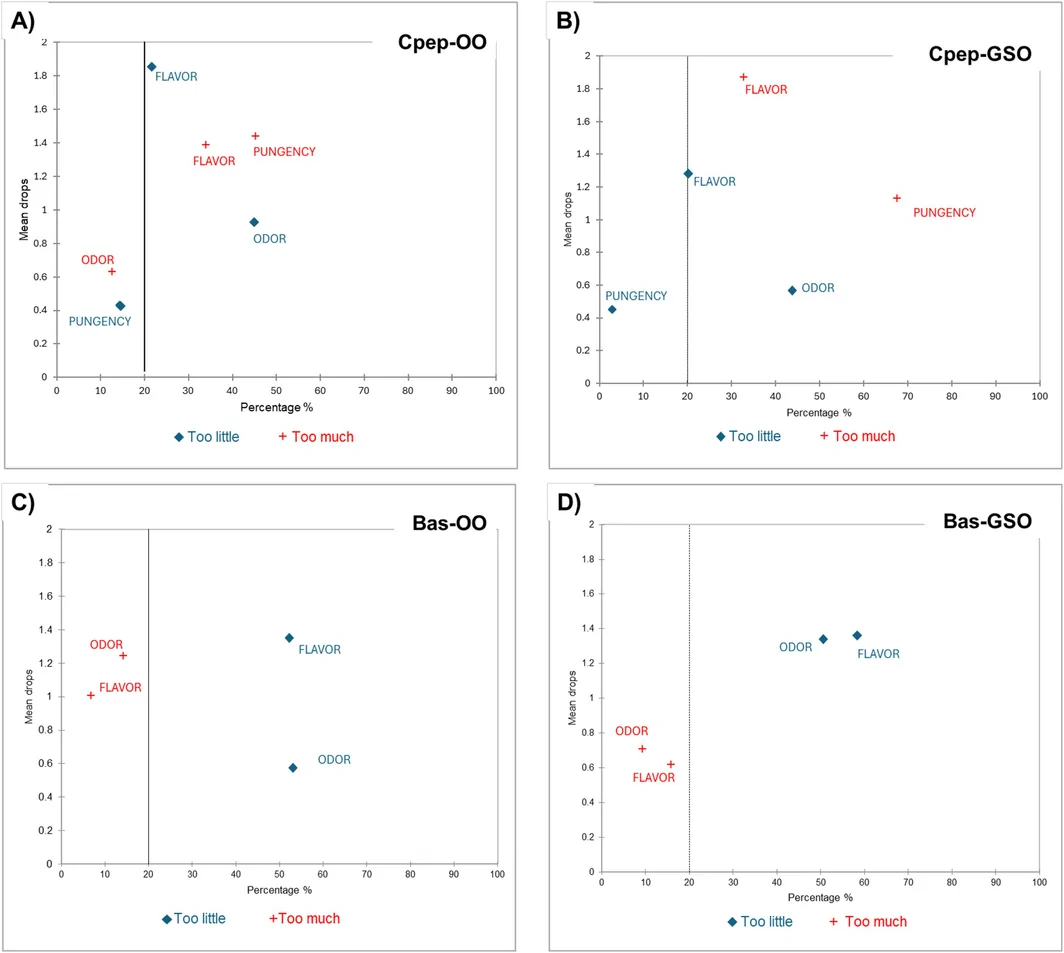
Graphs representing the mean drops of general liking vs the percentage of consumers expressing each attribute intensity. Chili pepper flavored olive oil (A); chili pepper flavored grapeseed oil (B); basil‐flavored olive oil (C); basil‐flavored grapeseed oil (D). The vertical line represents the 20% threshold considered.

Just‐about‐right (JAR) scales were applied to assess the adequacy of sensory attribute intensities and to identify potential areas for product optimization. The compressed bar charts in Figures S2 and S3 (for chili‐ and basil‐flavored oils, respectively) present the distribution of consumer responses across intensity categories. These data were subsequently used to perform penalty analysis, allowing the identification of attributes whose deviation from the JAR level was associated with significant decreases in overall liking (Gere 2022). The penalty analysis is an easy, powerful tool for consumer‐based food product development (Gere 2022; Zay and Gere 2019). Thus, for each one of the four oils, mean drops of general liking have been plotted against the percentage of consumers describing the attribute intensity as non‐JAR and being reported in the horizontal axis (Figure 3). Under blind conditions, the JAR‐based penalty analysis revealed attribute‐specific optimization needs that varied according to both flavoring matrix and oil. Attributes with more than 20% of responses in the “too little” or “too much” categories and mean drops exceeding 1 hedonic unit were considered critical reformulation targets.

For both chili‐flavored oils (Cpep‐OO and Cpep‐GSO), pungency and flavor were predominantly perceived as “too much”, with associated mean drops indicating a negative impact on overall liking, whereas odor showed a non‐negligible proportion of “too little” responses (Figure 3A,B). In particular, a more pronounced pattern was observed for Cpep‐GSO, where pungency exhibited the highest percentage of “too much” ratings by more than 60% of consumers, suggesting that excessive spiciness represented the main driver of liking reduction in this sample. The spiciness intensity was particularly underlined by consumers, even in “free description” questions about appreciated or unappreciated attributes (results in Figure S4), indicating the need for modulation of capsaicin‐derived intensity. Moreover, the panel consisted mainly of Italian participants. It is worth noting studies on the subjectivity of spiciness perception across populations and cultures, which may stem from varying degrees of sensitivity (Trachootham et al. 2018; Berry and Simons 2020).

Conversely, basil‐flavored oils showed a different trend. In Bas‐OO, flavor and odor intensities were mainly perceived as “too little”, with a substantial percentage of consumers (more than 50%) indicating insufficient aromatic intensity, highlighting the need for flavor enhancement. A comparable pattern emerged for Bas‐GSO, where both flavor and odor were frequently rated as “too little”, again associated with relevant liking penalties. Overall, the penalty analysis under blind conditions suggests that optimization strategies should focus on moderating pungency in chili‐flavored oils and increasing aromatic intensity in basil‐flavored samples, with relatively minor differences attributable to the kind of oil.

Along with the intensity parameters, data on the consumer willingness to repurchase the products was arranged and presented in Figure S5.

### 3.5 Influence of Information Disclosure on Consumer Liking and Perception of Basil‐Flavored Oils

**FIGURE 4:**
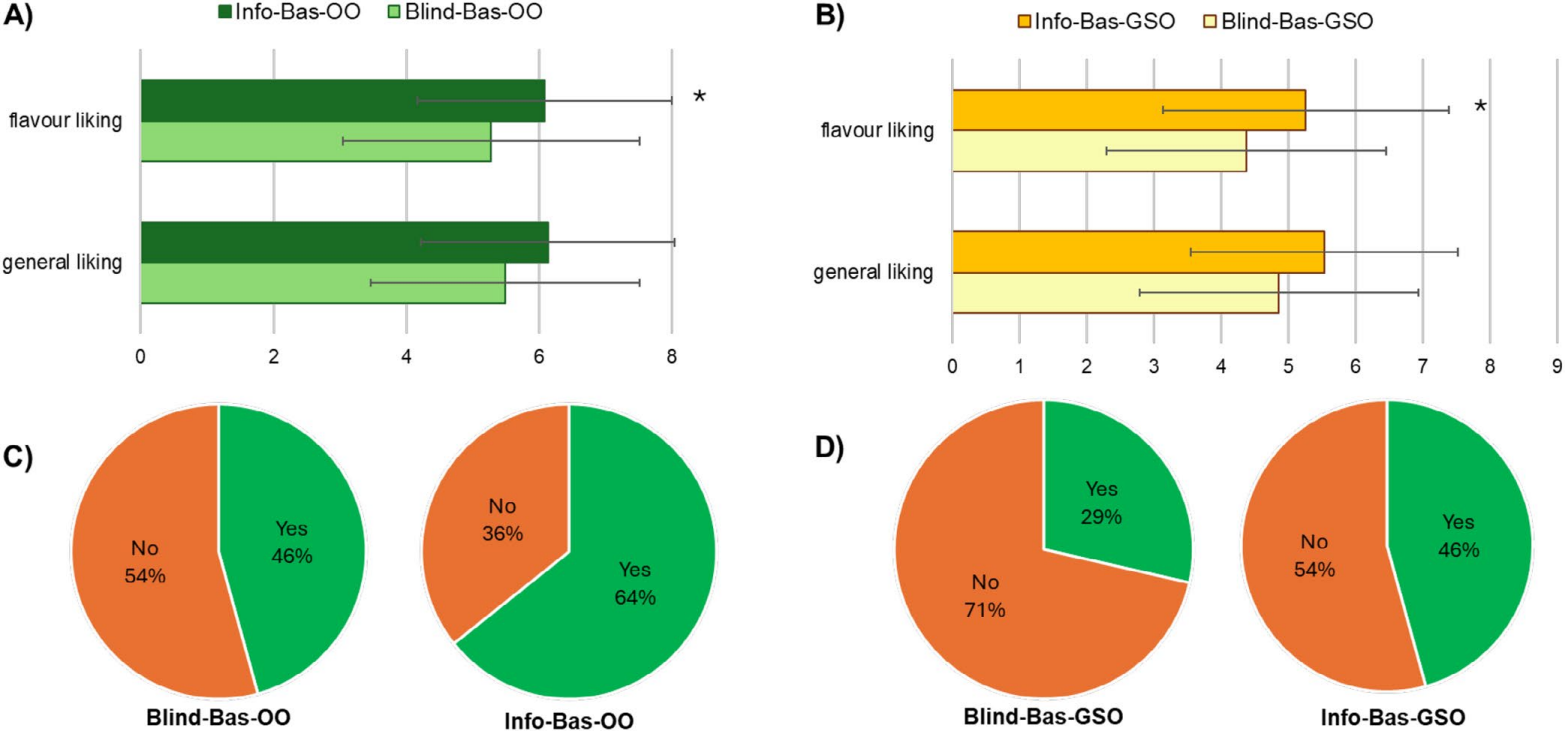
(A, B) Comparison of average flavor and general liking scores of basil‐flavored OO (A) or GSO (B) between blind and informed tests. The * indicates statistical differences between the results (one‐way ANOVA analysis followed by Tukey's HSD post hoc test). (C, D) Percentages of consumers’ willingness of repurchasing basil‐flavored OO (C) or GSO (D) according to the blind and informed tests.

Samples flavored with basil were subjected to an Informed Expectation test. Following the blind assessment of the intensity parameters, participants were given details on the oil type (OO or GSO), the aromatization matrix (basil), total polyphenol content, and antioxidant activity of the samples to examine how this information affected overall liking and flavor intensities scores. Providing information regarding functional enrichment and by‐product valorization significantly influenced consumer perception, particularly for basil‐flavored oils. Informed evaluation led to a statistically significant increase in flavor liking and overall appreciation compared to blind conditions, with the most pronounced effect observed for basil‐flavored GSO. This pattern is consistent with an assimilation effect, whereby positive expectations generated by health‐ and sustainability‐related information shifted hedonic judgments upward. The effect was more evident for the less familiar grape seed oil matrix, confirming that extrinsic cues may play a compensatory role when prior product knowledge is limited. As reported in Figure 4A,B, information induced a positive effect on flavor liking which significantly increased by 15.37% and 20.37% for Info‐Bas‐OO and Info‐Bas‐GSO, respectively, compared to the same samples tested in Blind (Blind‐Bas‐OO or Blind‐Bas‐GSO). The general liking enhancement was equal to 11.66% and 13.79% for Info‐Bas‐OO and Info‐Bas‐GSO respectively, even if not statistically significant. That suggests that consumer perceptions were positively influenced by information disclosure, but the magnitude of its impact can greatly vary depending on the product and attribute. Moreover, the information increased the willingness of repurchasing percentages from 46% to 64% for basil‐flavored olive oil and from 29% to 46% for grape seed oil (Figure 4C,D).

Along with the presented parameters, additional results sensory attributes of liking and disliking from the blind/informed basil‐flavored oils consumer tests comparison are reported in Figure S6. Among the freely indicated unpleasant sensory attributes, the “nothing” rises most frequently after the information received for Info‐Bas‐OO, with an increment of 19 consumers compared to the blind test. At the same time, in line with the observed increase in repurchase willingness, the proportion of consumers expressing positive appreciation increased for most sensory attributes, particularly for flavor and basil flavor in OO samples (Figure 4A).

The increase in hedonic scores under informed conditions was paralleled by a significant enhancement in purchase intention, particularly for basil‐flavored samples (Figure 4C,D). This finding indicates that expectation‐driven improvements in liking may translate into tangible market potential (Hurling and Shepherd 2003). Overall, the results demonstrate that consumer acceptance of flavored oils is shaped by a dynamic interaction between intrinsic sensory quality and extrinsic informational cues, and the differences observed between blind and informed conditions can be interpreted according to expectation–disconfirmation theory. That highlights how expectations can be developed by consumers on the basis of extrinsic product cues, and they are subsequently integrated into hedonic judgment and sensory evaluation (Sabbe et al. 2009). In the present study, information regarding polyphenol enrichment likely generated positive expectations related to healthiness and functional value, influencing consumers' liking scores even though the intrinsic sensory characteristics of the oils remained unchanged, and similar effects have been reported in the literature for nutritional and health‐related claims (Piqueras‐Fiszman and Spence 2015; Roselli et al. 2020). In particular, these findings further suggest how cognitive mechanisms play relevant roles in consumers' perception of flavored oils obtained through UAE: their sensorial experience was considerably associated with product information and technological innovation, even beyond sensory defects. The increase in liking observed under informed conditions suggests an assimilation effect driven by positive sustainability‐ and enrichment‐related expectations, particularly for the less familiar grape seed oil samples. While sensory optimization remains essential—especially for attributes identified through penalty analysis—strategic communication of functional and sustainability benefits may significantly enhance product positioning, particularly for underutilized oils such as grape seed oil. Anyway, considering that conducting blind and informed tests in the same session would have increased carry‐over and memory effects, that represents a limitation of this study, even if the experimental design aimed at minimizing these effects by using randomized blind sample presentation together with palate cleansing procedures (Lee and Kim 2013; Torrico et al. 2018). In addition, consumers were not informed that the same samples were evaluated again after information disclosure, reducing the likelihood of conscious memory‐driven responses. These findings suggest that information‐induced assimilation effects may not occur uniformly across all sensory dimensions, but rather depend on the interaction between product expectations and intrinsic sensory properties.

## 4 Conclusion

This study provides an integrated evaluation of the sustainable upgrading of underutilized refined olive oil and grape seed oil through ultrasound‐assisted extraction (UAE), combining chemical characterization with consumer perception under blind and informed conditions. UAE significantly increased the total phenolic and carotenoid contents of both oils, particularly in basil‐ and chili‐flavored formulations, respectively, without generally compromising their quality parameters. Nevertheless, the higher peroxide value observed in basil‐flavored grape seed oil indicates that oxidative changes were influenced more by the flavoring matrix than by the lipid carrier, highlighting the need to optimize processing and storage conditions for specific herb–oil combinations.

Consumer testing further demonstrated the potential of this approach. JAR and penalty analyses identified formulation improvements by increasing basil aroma intensity and moderating chili pungency, regardless of the oil matrix. Moreover, providing information on the enrichment process significantly enhanced consumer appreciation and purchase intention, particularly for the less familiar grape seed oil, supporting its valorization as a sustainable carrier of flavors and bioactive compounds. Although blind and informed evaluations were conducted within the same session, potential memory and carry‐over effects were minimized through randomized sample presentation and palate‐cleansing procedures.

Overall, these findings support UAE as a green technology for the simultaneous valorization of refined oils and food industry by‐products within a circular economy framework. However, the study was limited to the evaluation of overall bioactive enrichment through spectrophotometric assays, which provided suitable information for the informed consumer test but did not allow the characterization of individual compounds. In addition, long‐term storage stability, residual moisture content and particle size of the plant materials, and batch‐to‐batch variability were not investigated. Future research should therefore include targeted chromatographic and mass spectrometric analyses, complementary antioxidant assays (e.g., ABTS, FRAP, and ORAC), pigment and color stability evaluation, shelf‐life studies, and larger, more demographically diverse consumer panels to further support the industrial application of these sustainable flavored oils.

## Author Contributions

**Samanta Corsetti:** conceptualization, investigation, writing – original draft, methodology, formal analysis. **Lucia Irene Bailetti:** conceptualization, investigation, writing – original draft, validation, formal analysis, data curation. **Sofia Calzolari:** investigation, methodology, formal analysis. **Monia Floridi:** funding acquisition, resources, writing – review and editing. **Gianni Sagratini:** writing – review and editing, project administration, supervision, resources, funding acquisition, validation. **Laura Alessandroni:** conceptualization, visualization, software, methodology, writing – review and editing, investigation, formal analysis, data curation, supervision.

## Conflicts of Interest

The authors declare no conflicts of interest.

## Supporting information


**Table S1:** Questions, response scales and type of answers for all the basil and chili pepper‐flavored oils (section of Blind tests).
**Table S2:** Questions, response scales and types of answers of the intermediate section related to exploration of consumers awareness.
**Table S3:** Questions, response scales and types of answers of the last section related to the Informed test (Expectation test).
**Figure S1:** Histogram comparison showing the level of awareness of the consumers about the differences between EVO and OO (A), the knowledge about GSO (B) and the frequency of consumption of GSO, OO and EVO (C).
**Figure S2:** Bar charts of the frequencies of each rated categories and collapsed bar charts for Cpep‐OO (A) and Cpep‐GSO (B). Categories 1 and 2 are merged to the “too little” level, Category 3 becomes the JAR level, while Categories 4 and 5 are merged to the “too much” level in the collapsed graphs. Attributes investigated are the intensities of odor, flavor and pungency.
**Figure S3:** Bar charts of the frequencies of each rated categories and collapsed bar chart for Bas‐OO (A) and Bas‐GSO (B). Categories 1 and 2 are merged to the “too little” level, Category 3 becomes the JAR level, while Categories 4 and 5 are merged to the “too much” level. Attributes investigated are the intensities of odor and flavor.
**Figure S4:** Histogram comparison of specific attributes indicated by consumers as responses as those appreciated (Likes, A) or not (Dislikes, B) in chili pepper‐flavored oils (Blind test).
**Figure S5:** Percentages of consumers who would consume again chili pepper‐flavored OO (A) or GSO (B) (Blind test).
**Figure S6:** Histogram comparison of specific attributes indicated by consumers as those appreciated (A) or not (B) in basil‐flavored oils before and after receiving information on the product (Blind and Informed tests).

## Data Availability

The data that supports the findings of this study are available in the Supporting Information of this article.
