# Supplementary material for: From By‐Products to Flavored Oils: Ultrasound‐Assisted Enrichment of Refined Olive and Grape Seed Oils and Consumer Perception Under Blind and Informed Conditions
Source: Food Sci Nutr. 2026 Sep 17;14(9):e72374. doi: 10.1002/fsn3.72374 (PMC13586557; doi:10.1002/fsn3.72374)
Supplement: Supplementary file 1 [file FSN3-14-e72374-s001.docx]

# Supplementary material

**Table S1.** Questions, response scales and type of answers for all the basil and chili pepper-flavoured oils (section of Blind tests)

| Questions | Used scales or answers |
| --- | --- |
| How much do you like the colour of the oil? | 1-9 |
| How much do you like the product overall? | 1-9 |
| What do you particularly like about the product? | Short answer |
| What do you dislike? | Short answer |
| How much do you like the odour of the oil? | 1-9 |
| Intensity of the odour | 1-5 |
| How much do you like the flavour of the oil? | 1-9 |
| Flavour intensity | 1-5 |
| Would you consume again the product? | Yes or no |

**Table S2.** Questions, response scales and types of answers of the intermediate section related to exploration of consumers awareness

| Questions | Used scales or answers |
| --- | --- |
| How much grapeseed oil do you consume? | 1-5 (“not at all”, “a little”, “I consume it”, “a lot”, “very much”) |
| How much do you know about grapeseed oil and its nutritional properties? | 1-5 (“not at all”, “I've heard of it”, “I consume it”, “I consume it frequently”, “I always consume it”) |
| How much olive oil do you consume? | 1-5 (“not at all”, “a little”, “I consume it”, “a lot”, “very much”) |
| How much extra virgin olive oil do you consume? | 1-5 (“not at all”, “a little”, “I consume it”, “a lot”, “very much”) |
| Does extra virgin olive oil have the same nutritional properties as olive oil? | 1-4 (“certainly yes”, “probably yes”, “I don't know”, “no”) |

**Table S3.** Questions, response scales and types of answers of the last section related to the Informed test (Expectation test)

| Questions | Used scales or answers |
| --- | --- |
| How much do you like the product overall? | 1-9 |
| What do you particularly like about the product? | Short answer |
| What do you dislike about the product? | Short answer |
| How much do you like the flavour of oil? | 1-9 |
| Would you consume again the product? | Yes or no |

**Figure S1.** Histogram comparison showing the level of awareness of the consumers about the differences between EVO and OO (A), the knowledge about GSO (B) and the frequency of consumption of GSO, OO and EVO (C)

**Figure S2.** Bar charts of the frequencies of each rated categories and collapsed bar charts for Cpep-OO (A) and Cpep-GSO (B). Categories 1 and 2 are merged to the “too little” level, category 3 becomes the JAR level, while categories 4 and 5 are merged to the “too much” level in the collapsed graphs. Attributes investigated are the intensities of odour, flavour and pungency

**Figure S3.** Bar charts of the frequencies of each rated categories and collapsed bar chart for Bas-OO (A) and Bas-GSO (B). Categories 1 and 2 are merged to the “too little” level, category 3 becomes the JAR level, while categories 4 and 5 are merged to the “too much” level. Attributes investigated are the intensities of odour and flavour

**Figure S4**. Histogram comparison of specific attributes indicated by consumers as responses as those appreciated (Likes, A) or not (Dislikes, B) in chili pepper-flavoured oils (Blind test)

**Figure S5.** Percentages of consumers who would consume again chili pepper-flavoured OO (A) or GSO (B) (Blind test)

**Figure S6.** Histogram comparison of specific attributes indicated by consumers as those appreciated (A) or not (B) in basil-flavoured oils before and after receiving information on the product (Blind and Informed tests)
